# IgA/Kappa-restricted crystal storing histiocytosis involving the central nervous system characterized by proteomic analysis 

**DOI:** 10.5414/NP300645

**Published:** 2013-08-07

**Authors:** Bent A. Orr, Gary L. Gallia, Ahmed Dogan, Fausto J. Rodriguez

**Affiliations:** 1 Department of Pathology, St. Jude Children’s Research Hospital, Memphis, TN; 2 Department of Neurosurgery, The Johns Hopkins School of Medicine, Baltimore, MD,; 3 Department of Pathology and Laboratory Medicine, Mayo Clinic, Rochester, MN, and; 4 Department of Pathology, The Johns Hopkins School of Medicine, Baltimore, MD, USA

**Keywords:** crystal storing histiocytosis, brain tumor, plasma cell, mass spectrometry

## Abstract

Crystal storing histiocytosis (CSH) is a rare disorder characterized by the accumulation of crystalline material in the cytoplasm of histiocytes. Involvement of the central nervous system (CNS) with CSH is extremely rare. Herein, we report a case of crystal storing histiocytosis involving the CNS. Using immunohistochemistry and mass spectrometry we demonstrate that the disease resulted from an IgA-κ restricted plasma cell dyscrasia. CNS-CSH represents a rare clinicopathologic entity with an indolent course, usually lacking systemic manifestations.

## Introduction 

Crystal storing histiocytosis (CSH) is a rare disorder defined by the accumulation of crystalline material in the cytoplasm of histiocytes. Generally CSH is seen in the setting of a paraproteinemia either due to multiple myeloma, lymphoplasmacytic lymphoma, or monoclonal gammopathy of unknown significance [1, 2, 3]. Other rare, non-immmune causes of CSH include treatment with clofazimine for lepromatous leprosy, in association with Charcot-Leyden crystals, or due to silica exposure [4, 5, 6]. 

The immune-associated variant of CSH has been subgrouped into a generalized form involving two or more organs and a localized form involving a single organ site [7]. Generalized CSH most often involves the lymphoreticular system, bone marrow and kidney [7]. The organ sites most often affected by localized CSH include the lung and pleura, the orbit, the kidney and bone marrow (reviewed in [7]). Involvement of the CNS with CSH (CNS-CSH) is extremely rare. 

In this report, we describe a case of immune CSH involving the central nervous system in a 38-year-old woman with seizures. We used immunohistochemistry and mass spectrometry to determine that the CSH in this case was associated with an IgA-κ restricted plasma cell proliferation. To our knowledge, this is only the fourth instance in which CSH has been described in the CNS, and it is the first instance of CNS-CSH involving an IgA-restricted plasma cell proliferation. 

## Case history 

**Figure 1.**
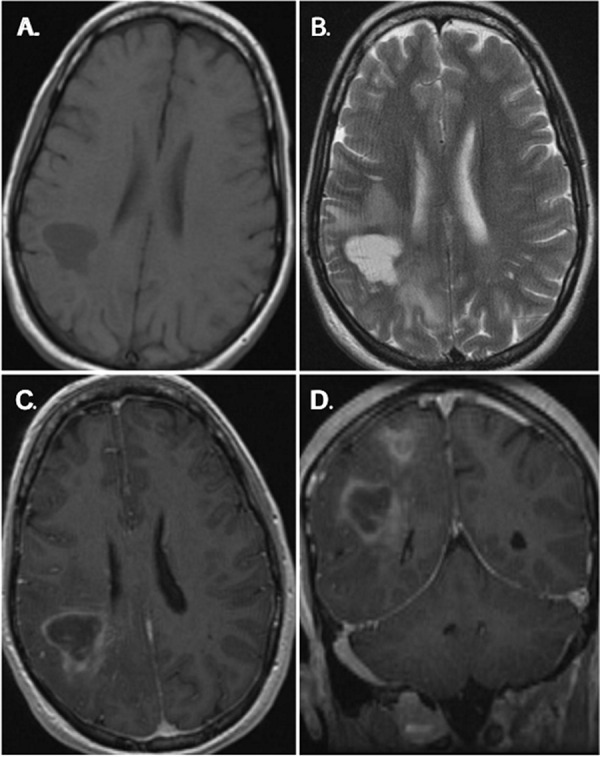
Magnetic resonance imaging (MRI) findings. Brain MRI demonstrates two irregularities. The primary lesion was centered in the posterior-inferior parietal lobe (A, B, C) and a second contiguous lesion was identified in the posterior-central parietal lobe (D). The lesions were T1-hypointense (A) and T2-hyperintense (B) with peripheral irregular enhancement on T1 imaging following administration of gadolinium (C, D). Abnormal T2/FLAIR hyperintense signal surrounded both lesions.

**Figure 2.**
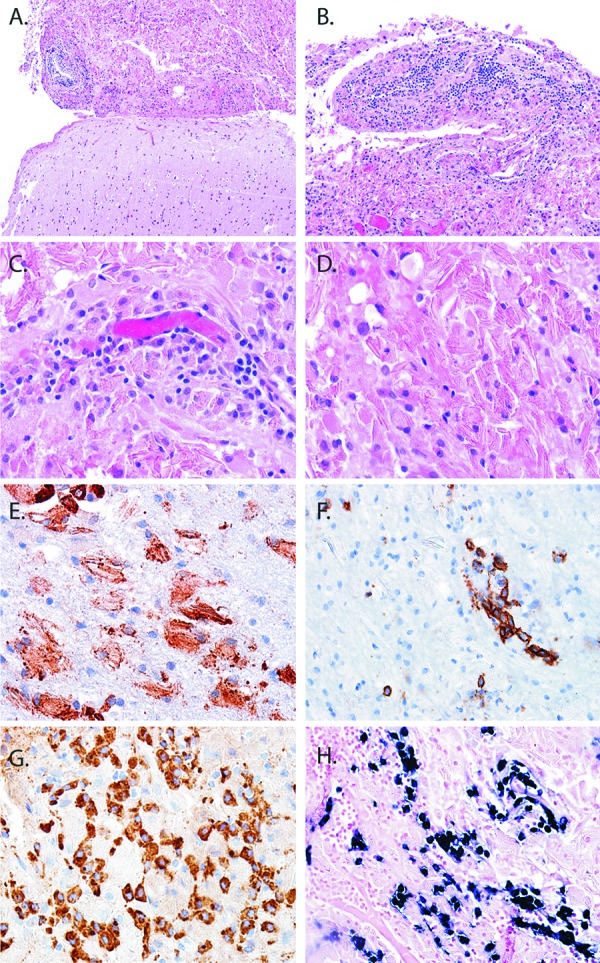
Pathology and immunophenotypic findings of CNS crystal-storing histiocytosis. A well circumscribed aggregate (top) was sharply demarcated from brain (bottom) (A). On higher magnification the infiltrate was composed of a mixed inflammatory infiltrate (B). A moderate number of plasma cells clustered around small blood vessels (C). Refractile, intracytoplasmic crystalline material was the hallmark feature of the lesion (D). Histiocytes expressed CD68 (E), while plasma cells expressed CD138 (F) and IgA by immunohistochemistry (G). In-situ hybridization studies for mRNA encoding immunoglobulin light chains showed κ-restriction (H).

The patient is a 38-year-old woman who presented with a tonic-clonic seizure in 2007. Cranial magnetic resonance imaging revealed a T1-hypointense (Figure 1A), T2-hyperintense lesion centered in the right parietal lobe (Figure 1). The lesion showed irregular areas of peripheral enhancement on T1 imaging with gadolinium (Figure 1C and Figure 1D). There was a second area of T2 hyperintensity with diffuse patchy enhancement superior to the main lesion (Figure 1D). Abnormal T2/FLAIR hyperintense signal surrounded both lesions. The patient also underwent MR spectroscopy imaging of the brain and the lesion demonstrated relative reduction in the NAA peak, as well as elevation of the choline, lactate and lipid peaks compared to normal brain parenchyma. A needle biopsy was performed. The patient’s seizures continued despite numerous medical therapies and she subsequently underwent a craniotomy in June of 2008 with biopsy. Postoperatively, the patient remained with seizures and was followed with serial imaging studies. On her August 2009 and August 2010 MRI scans, the more superior lesion increased in size. She subsequently sought an evaluation at our brain tumor center. On histologic examination, the lesion consisted of a hypercellular infiltrate effacing the normal brain parenchyma (Figure 2A). At low magnification the lesion was characterized by an intense chronic inflammatory infiltrate in a perivascular distribution (Figure 2B). On higher power the chronic inflammatory infiltrate was composed primarily of plasma cells (Figure 2C). The surrounding tissue contained abundant histiocytes. Within the histiocytes were needle-shaped eosinophilic cytoplasmic inclusions (Figure 2D). 

**Figure 3.**
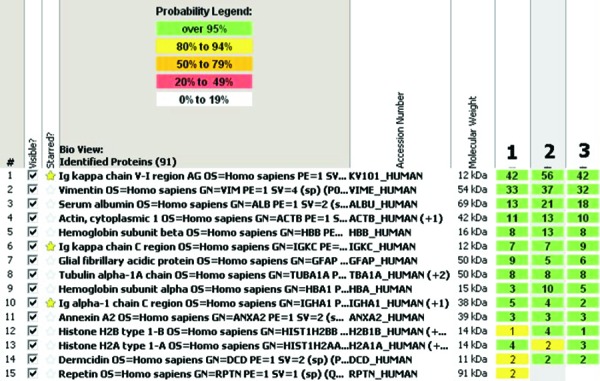
Mass spectrometry analysis with searches using previously published algorithms and probability score assignment revealed the abnormal infiltrates to be enriched by κ-light chains and IgA heavy chains (stars). Other peptides identified likely represent brain parenchyma components.

Lesional histiocytes were strongly immunoreactive for CD68 (Figure 2E). Immunohistochemical study for CD138 confirmed the plasma cell predominance in the perivascular infiltrates (Figure 2F). Further immunophenotyping of the plasma cell infiltrate revealed immunopositivity for IgA (Figure 2G). Using chromogenic *in situ* hybridization, the plasma cells were found to be Ig-ĸ-restricted (Figure 2H). To further characterize the nature of the crystals in an objective manner, a mass spectrometry proteomic based approach was performed. Proteomic studies of protein complexes, particularly those containing inmmunoglobulin deposits, have found increasing application in diagnostic surgical pathology. This is currently feasible in small tissue samples, and facilitated by enzymatic digestion, peptide separation by liquid chromatography and subsequent tandem mass spectrometry analysis. Following laser capture microdissection of the lesional tissue, mass spectrometry was performed on the formalin fixed and paraffin embedded tissue using previously published methods [8], confirming the presence of κ-light chain and the IgA constant region (Figure 3). Interestingly, there was especially high signal for the somatically mutated variable domain fragment of the immunoglobulin κ-light chain. Additional peptide fragments enriched in the lesion included glial fibrillary acid protein (GFAP) and multiple histone fragments which likely represent non-specific findings. GFAP is frequently identified in proteomic analysis of samples from the CNS, and in this instance may have been derived from associated reactive astrocytes. Given their abundance in all cell types, histone fragments are frequently identified in tissue samples from any source. Taken together the final diagnosis was crystal storing histiocytosis in association with an IgA-ĸ secreting plasma cell proliferation. 

The patient had additional workup performed. Her chest, abdominal and pelvic CT scans were unremarkable aside from a right ovarian cyst. A skeletal survey did not demonstrate any lytic lesions. A bone marrow biopsy revealed all normal hematopoietic elements. The patient was then treated with intensity-modified radiotherapy to the parietal lesions to a total dose of 36 Gy in 1.8 Gy fractions. Following radiotherapy she was treated with rituximab, 375 mg/m^2^ weekly for 4 weeks, then 375 mg/m^2^ once a month for 11 months and is currently on an every 3-month dosing regimen. Her brain MRI scans have remained stable over the past 17 months. 

## Discussion 

To our knowledge, the current case is only the fourth reported case of CSH involving the central nervous system (Table 1) [7, 8, 9, 10]. All four cases presented in young females with an age range of 27 – 38. The presenting symptoms in CNS-CSH have included focal neurologic deficits related to the site of the histiocytic collection, weakness or seizures (Table 1). In 2 of 4 cases, CSH was associated with an underlying plasma cell disorder [8, 10]. A single case was not associated with a lymphoproliferative disorder, but instead was reported in the setting of a patient with Crohn’s disease [9]. All reported cases of CNS-CSH showed a localized disease distribution with no evidence of systemic involvement or serum paraproteinemia. The disease presented in several neocortical locations, and in all cases involved the subcortical white matter. 

In the current case, imaging showed irregular nodular enhancement following administration of gadolinium with an associated increased intensity on T2-FLAIR. This is consistent with previous reports of CNS-CSH, in which magnetic resonance imaging has consistently shown irregular enhancement on T1-weighted images with contrast; however, both hypointensity and hyperintensity have been reported on T2-weighted images [9, 10]. Results from MR spectroscopy showed reduced NAA with an increase in the choline peak, consistent with previous reports [9]. By computed tomography, slight whiter matter attenuation and enhancement have been noted [9]. 

Generally Ig-κ is the predominant paraprotein involved in CSH [7]. It has been postulated that this is due to specific amino acid usage that promotes the accumulation of κ-light chain fragments by either enhancing secretion or reducing lysosomal degradation [2]. In the 3 cases of CNS-CSH in which the immunophenotype of the paraprotein was known, 2 cases involved Ig-κ [8]. In a single case the associated paraprotein was reported as polyclonal [9]. The current case is unique in that the IgA-restricted plasma cells were identified by immunohistochemistry and peptides from the IgA constant regions were identified by mass spectrometry. This is the first instance of an IgA-restricted plasma cell proliferation reported in association with CNS-CSH. IgA-restriction is uncommon in CSH at any location. For instance, Dogan et al. [7] reported that of the 37 cases in which heavy chain restriction was reported, only 16% (6/37) showed IgA-restriction. 

In two cases of CNS-CSH, including the current case, mass spectrometry was performed. In both cases, light chain variable fragments, a component of the light chain which undergoes somatic mutation, showed increased signal compared to the signal from constant region fragments. While the etiology of crystal storing histiocytosis is currently unclear, it is possible that specific somatic mutations in the variable regions predispose to crystal formation following posttranslational processing of immunoglobulins in macrophages. Supporting this etiology, Lebeau et al. [2] found unusual amino acid usage in the sequence of the variable region of an Ig-κ paraprotein in a case of generalized CSH. Additionally, a similar mechanism has been reported in human and animal models of other crystal forming diseases such as Fanconi’s syndrome [11, 12]. 

Given the limited accumulated clinical experience with the disease, the prognosis and optimal treatment of CSH is unclear, and many cases specific treatment depends on the associated underlying condition. Current data suggests that localized CSH has a better prognosis than generalized CSH [2]; however, even localized disease has not uniformly been controlled by chemotherapy or excision alone. For instance, Jones et al. [3] reported several examples of localized CSH showing persistent or recurrent disease following treatment with excision or chemotherapy. In the brain, the available data suggests that CNS-CSH may be a relatively indolent disease that can be controlled with treatment. For instance, in the three cases of CNS-CSH for which clinical follow up was reported (including the current case), the disease had either resolved or stabilized following therapeutic intervention. In 1 case, CNS-CSH showed dramatic improvement and achieved clinical remission with cyclophosphamide [13]. Similarly, Rodriguez et al. [8] reported clinical remission in a case of CNS-CSH after treatment with mephalan and blood brain barrier disruption with 25% intraarterial mannitol (two 10 mg/M2 treatments/month for 12 months) followed by low dose brain irradiation (3,600 cGy over 1 month). In the current case, the patient was treated with a combination of radiation and rituximab maintenance and showed clinical and radiographic stability over the 17 months since completion of her cranial radiotherapy. 

In summary, we report a rare case of crystal storing histiocytosis involving the central nervous system secondary to an IgA-restricted plasma cell dyscrasia. The disorder is rare but seems to behave in an indolent fashion in the brain, with a relatively favorable response to treatment. 

## Acknowledgments 

This work was partially funded by an NIH postdoctoral fellowship to B.A.O. (T32CA067751). 

**Table 1.** Clinical characteristics of reported cases of CNS-CSH.

Case	Age/Gender	Presentation	Location	Associated disorder	Paraprotein	Treatment	Follow-up
1^a^	27/F	focal neurologic deficits, tremor	white matter right parietal lobe	Crohn’s disease	unknown	unknown	unknown
2^b,c^	31/F	weakness, aphasia	bilateral white matter	monoclonal plasma cell proliferation	Ig-ĸ	lenolidamide/ dexamethasone; mephalan/radiation	progression at 1 year; stabilized following subsequent Tx
3^d^	38/F	seizures	blateral frontal lobe	unknown	polyclonal Ig	cyclophosphamide	responded well to Tx lesion resolved
4	38/F	seizures	white matter right parietal lobe	monoclonal plasma cell proliferation	Ig-ĸ/IgA	rituximab/radiation	responded well to Tx lesion for 17 months

^a^Kaminsky et al. [9]; ^b^Rodriguez et al. [8]; ^c^Costanzi et al. [10]; ^d^Prezeshkpour et al. [13] .
